# The most reactive iron and manganese complexes with *N*-pentadentate ligands for dioxygen activation—synthesis, characteristics, applications

**DOI:** 10.1007/s11144-021-02008-6

**Published:** 2021-06-15

**Authors:** Katarzyna Rydel-Ciszek

**Affiliations:** Department of Physical Chemistry, Faculty of Chemistry, Rzeszów University of Technology, al. Powstańców Warszawy 6, P.O. Box 85, 35-959 Rzeszów, Poland

**Keywords:** Bn-TPEN, Homogenous catalysis, Iron and manganese *N*-pentadentate catalysts, N4Py

## Abstract

The iron and manganese complexes that activate oxygen atom play multiple role in technologically relevant reactions as well as in biological transformations, in which exist in different redox states. Among them, high-valent oxo intermediate seems to be the most important one. Iron, and/or manganese-based processes have found application in many areas, starting from catalysis and sustainable technologies, through DNA oxidative cleavage, to new substances useful in chemotherapeutic drugs. This review is not only the latest detailed list of uses of homogeneous *N*-pentadentate iron and manganese catalysts for syntheses of valuable molecules with huge applications in green technologies, but also a kind of "a cookbook", collecting "recipes" for the discussed complexes, in which the sources necessary to obtain a full characterization of the compounds are presented. Following the catalytic activity of metalloenzymes, and taking into account the ubiquity of iron and manganese salts, which in combination with properly designed ligands may show similarity to natural systems, the discussed complexes can find application as new anti-cancer drugs. Also, owing to ability of oxygen atom to exchange in reaction with H_2_O, they can be successfully applied in photodriven reactions of water oxidation, as well as in chemically regenerated fuel cells as a redox catalyst.

**Graphical abstract:**

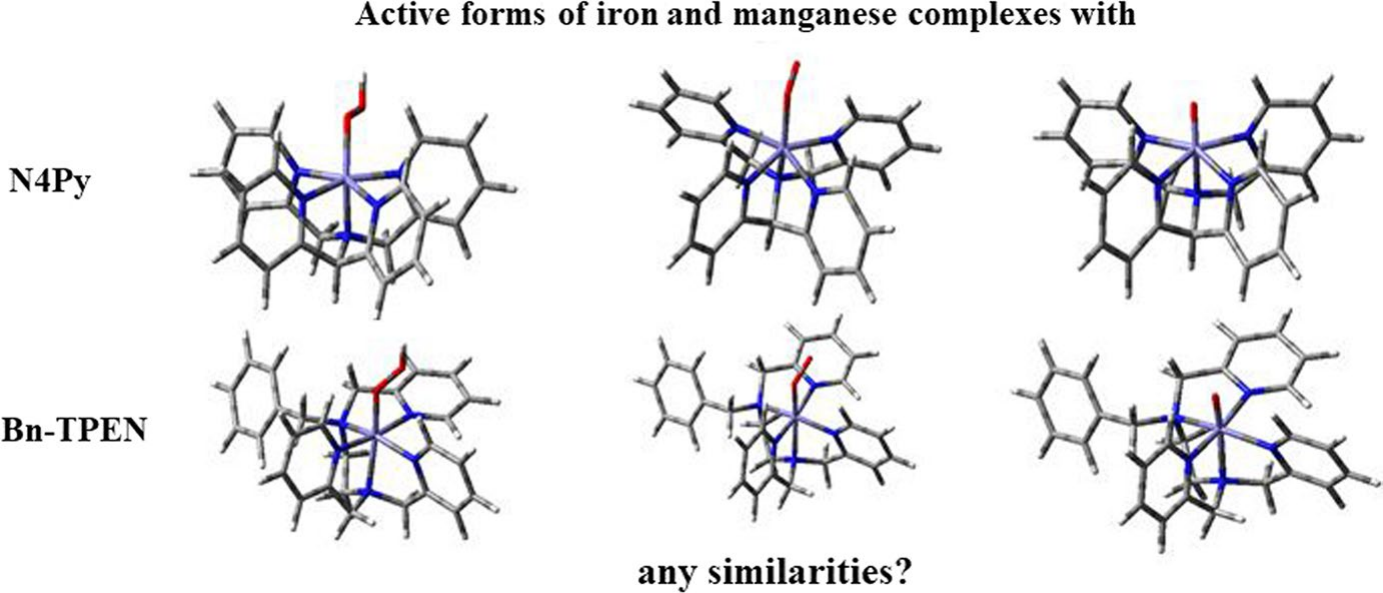

## Introduction

Mononuclear iron and manganese centers are common in oxygenases [1, 2]. Recently, synthesis of low-valent iron and manganese complexes capable of stabilizing intermediates produced in the oxygenation cycles, which show similarity to natural systems, is of great interest. Within the last two decades, a number of mononuclear complexes with oxoiron(IV) [3–20] and oxomanganese(IV) [9, 11, 18, 21] units supported by polydentate non-heme ligands have been investigated. These complexes serve as models for high-valent intermediates in the catalytic cycles of enzymes that carry out a series of oxidative modifications. Iron(IV)-oxo complexes as active oxidants were recognized in: α-ketoglutarate dioxygenase, halogenase [22–25], and/or methane monooxygenase [22, 23]. These intermediates were identified with different spectroscopic techniques, on the basis of which it was founded that, they have a high-spin [spin (*S*) = 2] iron(IV)-oxo centre and the bond between the oxygen atom and iron ion shows double bond feature [24]. Furthermore, high-valent iron and manganese oxo complexes, produced in the reaction of water oxidation, were determining species in the "oxygen-evolving complex" (OEC) in photosystem II [4, 7, 26]. Manganese also plays an important role in many biological processes in which, similarly to iron, is present in different oxidation states. In organisms, the manganese-containing ribonucleotide reductases catalyze the transformation of ribonucleotides to deoxyribonucleotides, which is a preliminary step in DNA repair and replication [27]. Following the structure of enzymes, in laboratory conditions, complexes imitating the structure of their active centers were synthesized. Particularly effective for this purpose are ligands constructed with a fivefold pyridine donor set. Pentadentate ligands can be assigned into eight subgroups, this division is related with the binding each of the five *N-*donors to the central atom [28]. Based on article [28], there are known: ligands with linear network of five nitrogen atoms, tetracyclic ligands with a linked one *N*-donor side ring, tricyclic ligands with linked two *N*-donor side rings (three subgroups related with the position of two "pendant" donors), tetrapodal ligands, such as N4Py, elongated tripodal ligands like TPMEN, and moreover macrocyclic ligands [28–30]. Iron and manganese complexes containing pentadentate ligands are also broadly used as oxidants in many areas, e.g. biotechnology, synthesis of organic compounds, transformation of organic compounds, pharmacy as components that react with DNA [31–35]. These complexes have been successfully used as photosensitizers in solar cells and in redox and catalytic light-controlled processes.

## *N*-pentadentate non-heme iron and manganese complexes characterization

**Fig. 1:**
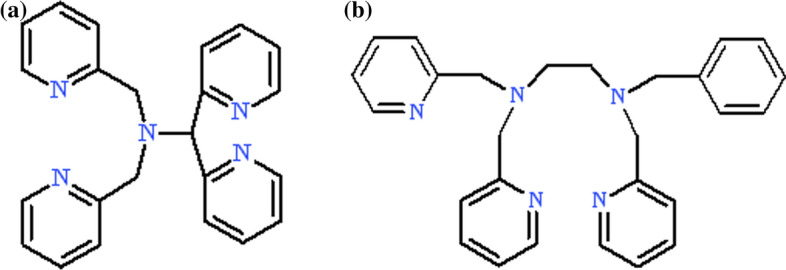
Structural formulas of *N*-pentadentate ligands discussed in this work, **a** N4Py, **b** Bn-TPEN (hydrogen atoms were omitted)

Iron complex with N4Py ligand (Fig. 1a) and its oxygen adducts are perhaps the most extensively studied of the non-heme complexes. In 1999, Feringa, Que et al*.* [36] investigated a non-heme mononuclear iron(II) complex with N4Py ligand {NMR data – [37]} to gain more information about the formation and stability of non-heme iron(III) hydroperoxo intermediates.

[(N4Py)Fe^II^(MeCN)]^2+^ (**1**) is formed as a dark red crystalline solid, with high yield in reaction of N4Py and Fe(ClO_3_) [as well as Fe(ClO_2_)], in a methanol/acetonitrile solution, and was characterized by ^1^H NMR, UV/Vis [37], ES/MS [30] techniques, moreover iron-N4Py complex can also be obtained by reacting with another iron salt – Fe(OTf)_2_·2MeCN [17]. Cyclic voltamperograms of (**1**) indicates a reversible oxidation peak [at 0.99 V (*vs*. SCE)] associated with the Fe^II^/Fe^III^ couple, and probably presenting the thermodynamic stability of the iron(II) complex [37]. The X-ray structure of this complex specify that iron centre is a six-coordinate ones; the *N*-pentadentate ligand saturates five iron coordination sites, leaving one free binding site to an acetonitrile molecule, which can simply be displaced by, for example, dioxygen. The [(N4Py)Fe^II^]^2+^ complex, due to the similarity in structure, is known as a synthetic model for iron bleomycin (BLM), on the other hand the BLM ligand, has been proposed to be an intermediate between heme and non-heme chemistry [38]. Iron-bleomycin, metalloglycopeptide clinically used in anti-cancer therapy [39–41], in the presence of dioxygen can trigger selective oxidative DNA cleavage [31, 33, 35, 42, 43]. The antitumor drug BLM is proposed to act via a low-spin iron(III) hydroperoxide intermediate called "activated bleomycin" [44]. Iron(II) complex with N4Py (**1**) due to the short Fe–N bond with a length of 1.91–1.96 Å is a low-spin (LS) one [37]. In reaction with H_2_O_2_ it turns into purple compound [(N4Py)Fe^III^(OOH)]^2+^ (**2**) named "purple intermediate" [37], which is capable of oxidizing various organic substrates [28, 44–47]. However, formation of (**2**) is closely dependent on used solvent e.g. in acetonitrile, the [(N4Py)Fe^III^(OOH)]^2+^ complex is obtained only in huge excess (> 50 equiv) of hydrogen peroxide, while in methanol, reaction with H_2_O_2_ is very fast, and only 1.2–2 equivalents of H_2_O_2_ are required to produce (**2**) [47]. Intermediate (**2**) (specified by EPR spectra, where *g* = 2.17, 2.12, 1.98 [37]) have also low-spin iron(III) centers (*S* = 1/2), showing charge-transfer bands near the 500–600-nm region related with transition hydroperoxo-to-iron(III). Their ν_O-O_ frequencies occurring near 800 cm^−1^ in the Raman spectrum, are lower than those obtained for their high-spin (HS) analogues [30]. (**2**) was also analyzed with techniques such as Raman {bands at 632 cm^−1^ (corresponding to strong Fe–O bond) and ~ 790 cm^−1^ (related with weak O–O bond) [30, 38, 45]}, ES/MS {*m/z* = 555 – {[(N4Py)Fe(OOH)]ClO_4_}^+^; *m/z* = 753 – {[(N4Py)Fe(OOH)](ClO_4_)_3_}^−^ [36, 37]}, or by the use of DFT calculations {average of distances: *r*(Fe–O): 1.804, *r*(O–O): 1.490, *r*(Fe-N_tr_): 2.033, *r*(Fe-N_c_): 1.996 [45]}. The formation of (**2**) undergoes via two stages. In the first one, the addition of hydrogen peroxide to acetonitrile solution containing (**1**) causes the formation of [(N4Py)Fe^III^-OH]^2+^ complex (*g* = 2.41, 2.15, 1.92 specified by EPR spectra [30]); in the second stage – hydroperoxide group from H_2_O_2_ takes the place of hydroxide to form (**2**) [30, 38, 48]. [(N4Py)Fe^III^(*η*^1^-OOH)]^2+^ can be converted to its conjugate base called "blue intermediate", which is a high-spin iron(III) adduct (with *S* = 5/2), in which the peroxo group is a side ligand, [(N4Py)Fe^III^(*η*^2^-O_2_)]^+^ [30, 49]. In Raman spectroscopy HS iron(III)-peroxo complexes, often show bands at 400–500 cm^−1^ region and in the 815–900 cm^−1^ area singed to *ν*_Fe-O_ and *ν*_O-O_ features, respectively. [(N4Py)Fe^III^(*η*^2^-O_2_)]^+^ is characterized by Raman (495 and 827 cm^−1^), UV/Vis [methanol λ_max_ (ɛ) = 685 nm (520 M^−1^· cm^−1^)], EPR (*g* = 8.00, 5.60, 4.30) and MS (*m*/*z* = 455 – {[(N4Py)Fe(OO)]}^+^) spectroscopy; also DFT geometry optimization was used for this complex and metal–ligand distances (Å) have been defined as: Fe–O, 1.96; Fe-N_py_, 2.17 and 2.27, Fe-N_amine_, 2.39 [30]. Unfortunately, [(N4Py)Fe^III^(*η*^2^-O_2_)]^+^ is unable to catalyze the hydrogen atom transfer (HAT) reaction [30]. In contrast, the "purple intermediate" (**2**) is capable of HAT reaction and has been proposed to be a precursor in the catalytic cycle for the generation of high-valent intermediates [36]. Raman studies of [(N4Py)Fe^III^(OOH)]^2+^ indicated a weakened O–O bond in the complex, so homolysis of the O–O bond of (**2**) can produce [(N4Py)Fe^IV^ = O]^2+^ (**3**) intermediate [30, 38, 45]. Additionally, the green complex (**3**), oxo type, can be generated at room temperature in reaction with an excess of peracid or iodosylbenzene (PhIO), displaying considerable thermal stability and its shelf life is in the order of several days (*t*_1/2_ = 60 h) [50, 51]. The stability of iron(IV)-oxo species is dependent on the pH of reaction solutions, complex (**3**) is stable at low pH (in the region of 5–6) but it decays rapidly as the pH of the reaction solutions increases [52]. Thus, (**3**) belongs to an rising class of Fe^IV^ = O non-heme complexes, with a near-IR spectral signature [optical d-d transition between 800 nm and 900 nm, related to green colour of (**3**)], as well as Raman spectra between 750 cm^−1^ and 850 cm^−1^ [13, 51], with a short Fe–O distance of ~ 1.64 Å [50], which makes it similar to oxoiron(IV) porphyrin species [53]. The oxidation state of the iron central atom can be determined by the use of advanced techniques such as Mössbauer, X-ray absorption near edge spectroscopy (XANES) [22, 24] or/and ^1^H NMR [50]. The high oxidative power of (**3**) was suggested not only by catalytic research [54, 55] but also by theoretical studies [56, 57]. Based on density functional theory (DFT) calculations, it was found, that in case of complexes of iron(IV)-oxo, those with *S* = 2 have π * and σ * paths enabling the oxidation of the C-H bond, and complexes with *S* = 1, in this reaction, can only use the π * path [58]. These paths have some geometrical limitations – the σ* path is the most favorable when C-H bond, of organic compounds, approaches Fe = O species along the Fe–O axis, and the π* path is the most possible when C-H is upcoming under 120° with respect to Fe = O center [58]. The DFT calculations of reaction of cyclohexane hydroxylation by the complex [(N4Py)Fe^IV^ = O]^2+^ were performed [56]. The triplet is the ground state, but it is also possible for this complex to occur in both a quintet and a singlet states [56]. Based on the DFT calculations, in which the reaction of oxidation of cyclohexene to alcohol catalyzed with a non-heme complex (**3**) was analyzed, Shaik et al*.* comparing [(N4Py)Fe^IV^ = O]^2+^ with Cpd I cytochrome P450, showed that (**3**) is more reactive and exhibit a distinct solvent effect [56]. Additionally, some information regarding oxidation capacity of Fe^IV^ = O complexes were obtained on the basis of electrochemical research. Collins, Que et al*.* carried out bulk electrolysis in aqueous/MeCN to generate (**3**) from its iron(II) precursor [6]. Electrolysis at potentials above + 0.61 V generated [(N4Py)Fe^III^(OH)]^2+^, (UV/Vis – yellow chromophore, *λ*_max_ = 320 nm), at higher potentials the yellow solution was converted to the green one (*λ*_max_ = 695 nm) characteristic to (**3**) [6]. This positive redox potential explains the greater alkane oxidation reactivity of [(N4Py)Fe^IV^ = O]^2+^ [22].

The Bn-TPEN (*N*-benzyl-*N*,’*N’*,*N*’-tris(2-pyridylmethyl)-1,2-diaminoethane) {NMR data – [59]} is also *N*-pentadentate ligand (Fig. 1b), where individual proportions of pyridine and amine groups allow for precise adjustment of properties and stability of iron complexes, and its [(Bn-TPEN)Fe^II^]^2+^ (**1′**) complex is very similar to (**1**). [(Bn-TPEN)Fe^II^(OTf)](OTf) is formed with high yield as a yellow powder in a reaction of Bn-TPEN ligand with Fe(OTf)_2_·2MeCN in a dichloromethane solution [17, 51]. There is also known the reaction of Bn-TPEN with either iron(II) or iron(III) chloride in dry methanol, followed by addition of a salt of the counter anion (ClO_4_^−^), leading to precipitation of yellow [(Bn-TPEN)FeCl]ClO_4_ and [(Bn-TPEN)FeCl](ClO_4_)_2_ complexes [59]. It is interesting that, in the case of iron(II) complexes their spin states are closely related to the solvent used for the research. In acetonitrile, low-spin iron(II) complexes are preferentially formed, and high-spin ones – in acetone [36]. This observations was confirmed by ^1^H NMR spectroscopy [60]. Measurements performed using cyclic voltammetry as a technique, indicated that for iron(II) complexes their redox potentials Fe^III/II^ for those with LS are much higher, than for those with HS. In MeCN solutions the iron(II) complexes reveal reversible redox behaviour, while in MeOH – irreversible. In acetonitrile higher potentials of iron(II) complexes LS are observed [*E*^0'^ = 1.00 V for (**1**) and 0.95 V and for (**1′**) *vs* SCE], whereas in methanol for HS – lower ones [*E*_pc_ = 0.29 V, *E*_pa_ = 0.63 V for (**1**) and *E*_pc_ = 0.29 V, *E*_pa_ = 0.75 V for (**1′**) *vs* SCE] [60]. It is very important that the solvent (e.g. alcohol) plays an invaluable role in the formation of iron(III) complexes type hydroperoxo, both in the case of complexes (**1′**) and (**1**). Both these complexes can be used for dioxygen activation. In first step of this reaction, the high-spin iron(II) complex reacts with dioxygen to form an iron(III)-superoxo adduct, which in further transformations gives the low-spin hydroperoxo complex of iron(III) [60]. The coordinative saturated *S* = 1/2 [(Bn-TPEN)Fe^III^(OOH)]^2+^ (**2′**) species, can be produced, also in the reaction with excess hydrogen peroxide in hydroxylic solvents in the form of purple species stable for hours, named "sluggish oxidant" that undergoes via homolysis of the O–O bond [3]. Formation of (**2′**) in the presence of H_2_O_2_ occurs, analogous to (**2**), in two stages. Jensen et al*.* [59] assigned peaks of purple solutions observed in the ESI mass spectra to [(Bn-TPEN)Fe^III^(OOH)]^2+^ and [(Bn-TPEN)Fe(OO)]^+^ complexes. In the spectra of "blue solutions", an intense *m/z* signal at 256 and a weak signal at *m/z* 511 were obtained for the acid–base equilibrium. Usually the most intense peak belongs to the ferryl species of [(Bn-TPEN)Fe^IV^ = O]^2+^ complex. Collision activated dissociation (CAD) of the ion at *m/z* 256 gives ions at *m/z* 248, 240 and 195, assigned to respectively ferryl species [(Bn-TPEN)Fe^IV^ = O]^2+^, ferrous species [(Bn-TPEN)Fe^II^]^2+^, and to [(Bn-TPEN)Fe^II^-C_7_H_6_)]^2+^ [59]. EPR analysis confirmed (**2′**) as low-spin Fe(III) complex – [(Bn-TPEN)Fe^III^(*η*^1^-OOH)]^2+^ (*g* = 2.20, 2.16, 1.96). After addition of base to high-spin iron(III) blue solutions of [(Bn-TPEN)Fe^III^(*η*^2^-O_2_)]^+^ new signals (at *g* = 7.60 and 5.74) is produced [59]. Treatment of (**1′**) with excess solid PhIO in MeCN at 25 °C provides a green compound [(Bn-TPEN)Fe^IV^ = O]^2+^ (**3′**) with *λ*_max_ at 739 nm and a shoulder near 900 nm, but this species is less stable (about ten times) than (**3**) (*t*_1/2_ = 6 h) [17, 51]. Monocrystals of (**3′**) have not been separated yet, therefore information on its structure should be obtained by alternative methods [50]. Complex (**3′**) was characterised by ES/MS spectroscopy [51, 59], X-ray crystallography [22, 50] or EXAFS analysis [22]. The ligand flexibility indicates the possibility of the formation of three isomers: A, B and C—differing in the position and number of pyridine rings, at a given position relative to the Fe–O axis (pyridine rings can eclipse Fe–O axis, can be perpendicular to the axis, or can coordinate to the oxygen atom in the trans position) [50]. Analysis of the ^1^H NMR spectrum of (**3′**) implies that the Bn-TPEN ligand in complex with iron ions has an A-isomer structure—with one pyridine ring located perpendicular to the Fe–O axis and two pyridine rings eclipse on this axis [50]. Those observation were also confirmed by theoretical research. Considering the DFT calculations of (**3′**), isomer A was found to be the most stable and the average distance of each Fe–N bond was approximately 2.040 Å. Comparing this catalyst with an analogous complex but build with the N4Py ligand, which in turn obtains excellent stabilization because, it has four *N*-rings situated parallel to the Fe = O line (the average Fe–N bond distance is 2.00 Å), explains why half-life of (**3**) is longer than (**3’**) [50]. Based on electrochemical measurements, the half-wave potentials of (**3’**) was determined as one-electron reduction potentials equal to 0.49 V *vs* SCE. This approach allows to understand the mechanism of the demonstrated catalytic activity, assuming the possibility of electron transfer (ET) reactions of iron(IV)-oxo complexes with N4Py and Bn-TPEN, originally adopted by Lee et al*.* [61]. They demonstrated that the reorganization energies of the electron-transfer (ET) reduction of complexes (**3’**) and (**3**) increase (Bn-TPEN, 2.55 eV < N4Py, 2.74 eV), when one-electron reduction potentials (E_red_) is shift in positive site (Bn-TPEN, 0.49 V < N4Py, 0.51 V *vs* SCE). It can be assumed that the higher E_red_ of Fe^IV^ = O complexes is achieved by compensating for the greater reorganization energy, which includes the corresponding structural change needed for the electron relocate reaction. Flexible non-heme ligand compared to the inflexible heme ligand allows for greater reorganization of the bond after ET reduction, which may be connected with higher reorganization energies values [61].

Steric and electronic aspects of the ligands N4Py and Bn-TPEN, and their complexes with iron, along with the fact of low toxicity of manganese compounds compared to iron [62], encouraged me to focus my attention on this kind of manganese complexes with *N*-pentadentate ligands. Table 1 presents a selected spectroscopic properties of both iron and manganese complexes.

**Table 1:** Selected spectroscopic properties of iron and manganese complexes with N4Py or/and Bn-TPEN ligands

Complexes	UV/Vis	References	EPR	References	ES-MS	References
[(N4Py)Fe^II^]^2+^	Acetone: λ_max_ (ɛ) = 382 nm (5700 M^−1^·cm^−1^), 458 nm (4000 M^−1^·cm^−1^)	[37]	EPR-silent	[33, 36]	*m*/*z* = 520 {[(N4Py)Fe^II^](ClO_4_)(MeCN)}^+^ *m*/*z* = 210 {[(N4Py)Fe^II^](2ClO_4_)(MeCN)}^2+^ *m*/*z* = 522 {[(N4Py)Fe^II^](ClO_4_)}^+^	[30] [33]
[(N4Py)Fe^III^(OOH)]^2+^	Acetone: λ_max_ (ɛ) = 530 nm (1100 M^−1^·cm^−1^) MeOH: λ_max_ (ɛ) = 548 nm (1100 M^−1^·cm^−1^)	[36–38] [36, 45]	*g* = 2.17, 2.12, 1.98	[36, 37]	*m*/*z* = 555 {[(N4Py)Fe^III^(OOH)](ClO_4_)}^+^ *m/z* = 753 {[(N4Py)Fe^III^(OOH)](ClO_4_)_3_}^−^	[30] [36, 37]
[(N4Py)Fe^III^(OH)]^2+^	MeCN: *λ*_max_ = ~ 320 nm	[6]	*g* = 2.41, 2.15, 1.92	[36]	*m/z* = 539 {[(N4Py)Fe^III^(OH)](ClO_4_)}^+^	[33]
[(N4Py)Fe^III^(*η*^2^-O_2_)]^+^	MeOH: λ_max_ (ɛ) = 685 nm (520 M^−1^·cm^−1^)	[30]	*g* = 8.00, 5.60, 4.30	[30]	*m*/*z* = 455 [(N4Py)Fe^III^(OO)]^+^	[30]
[(N4Py)Fe^VI^ = O]^2+^	MeCN: λ_max_ (ɛ) = 695 nm (400 M^−1^·cm^−1^)	[6, 17] [51]	EPR-silent	[4]	*m/z* = 221 [(N4Py)Fe^VI^ = O]^2+^ *m/z* = 538{[(N4Py)Fe^VI^ = O](ClO_4_)}^+^	[7] [7, 51]
[(Bn-TPEN)Fe^II^]^2+^	MeCN: λ_max_ (ɛ) = 322 nm (800 M^−1^·cm^−1^), 401 nm (2050 M^−1^·cm^−1^), 894 nm (17 M^−1^·cm^−1^)	[49]	EPR-silent	[17, 49]	*m/z* = 240 [(Bn-TPEN)Fe^II^]^2+^ *m/z* = 628 {[(Bn-TPEN)Fe^II^](OTf)}^+^	[49, 59] [51]
[(Bn-TPEN)Fe^III^(OOH)]^2+^	MeCN: *λ*_max_ = 542 nm	[49]	*g* = 2.20, 2.16, 1.96	[49, 59]	*m/z* = 256 [(Bn-TPEN)Fe^III^(OOH)]^2+^	[59]
[(Bn-TPEN)Fe^III^(*η*^2^-O_2_)]^+^	MeCN: λ_max_ = 770 nm	[49]	*g* = 7.60, 5.74	[49, 59]	*m/z* = 511 [(Bn-TPEN)Fe^III^(OO)]^+^	[49, 59]
[(Bn-TPEN)Fe^VI^ = O]^2+^	MeCN: *λ*_max_ (ɛ) = 739 nm (400 M^−1^·cm^−1^)	[17, 51]	EPR-silent	[17]	*m/z* = ~ 249 [(Bn-TPEN)Fe^VI^ = O]^2+^ *m/z* = 644 {[(Bn-TPEN)Fe^VI^ = O](OTf)}^+^	[7, 59] [51]
[(N4Py)Mn^II^]^2+^	Acetate buffer: λ_max_ (ɛ) = ~ 260 nm (154 M^−1^·cm^−1^)	[26]	EPR-silent	[71]	*m/z* = 457 {[(N4Py)Mn^II^](Cl)}^+^	[64]
[(N4Py)Mn^VI^ = O]^2+^	CF_3_CH_2_OH-MeCN: λ_max_ = 940 nm CF_3_CH_2_OH: λ_max_ (ɛ) = ~ 940 nm (≈ 250 M^−1^·cm^−1^)	[71] [63, 67]	*g* = 5.80, 3.20, 2.01	[71]	*m*/*z* = 220 [(N4Py)Mn^VI^ = O]^2+^ *m*/*z* = 240 {[(N4Py)Mn^VI^ = O](MeCN)}^2+^ *m*/*z* = 587 {[(N4Py)Mn^IV^ = O](OTf)}^+^	[63]
[(Bn-TPEN)Mn^II^]^2+^	Acetate buffer: λ_max_ = ~ 260 nm MeCN/MeOH: λ_max_ = ~ 260 nm	[26] [84]	*g* = 5.36, 3.60, 2.06	[69]	*m*/*z* = 239 [(Bn-TPEN)Mn^II^]^2+^ *m*/*z* = 627 {[(Bn-TPEN)Mn^II^](OTf)}^+^	[69]
[(Bn-TPEN)Mn^VI^ = O]^2+^	CF_3_CH_2_OH: λ_max_ (ɛ) = ~ 1040 nm (220 M^−1^·cm^−1^)	[69]	*g* = 5.53, 2.76, 1.76	[69]	*m*/*z* = 247 [(Bn-TPEN)Mn^VI^ = O]^2+^ *m*/*z* = 643 {[(Bn-TPEN)Mn^IV^ = O](OTf)}^+^	[69]

Yellow microcrystals of the complex [(N4Py)Mn^II^]^2+^ (**4**) were obtained by the reaction of the Mn^II^(OTf)_2_ salts and N4Py ligand in acetonitrile under an Ar atmosphere [26, 63]. The complex was analysed with mass spectrometry using ESI (ESI–MS), UV/Vis spectroscopy, along with the X-ray diffraction (XRD) technique or DFT calculations [26, 63].

It was reported that peroxomanganese(III) adduct can be produced both in reaction with KO_2_ and H_2_O_2_, so for generation of [(N4Py)Mn^III^(O_2_)]^+^ complexes the excess of KO_2_ in MeCN solutions was used [64]. A "blue solution" with a prominent peak at *m/z* 454 in an ESI–MS experiment, consistent with its formation as [(N4Py)Mn^III^(O_2_)]^+^ was obtained [64]. Kaizer et al*.* [65] presented that using water as the reaction environment, non-heme manganese(IV)-oxo complexes showed similarity to the reaction of catalase. N4Py^*^ manganese(II) ion complexes, similar to [(N4Py)Fe^II^]^2+^, can directly react with hydrogen peroxide to give Mn^III^OOH, and then the homolytic cleavage of O–O results in the formation of manganese(IV)-oxo compound [65]. The oxidative reactivity of manganese hydroperoxides, during the reaction with organic substrates was also investigated [66], but so far complexes such as Mn^III^OOH with N4Py and Bn-TPEN ligands are hardly characterized in literature.

As is the case of iron complexes with N4Py, the formations of a greenish-yellow [(N4Py)Mn^IV^ = O]^2+^ species (**5**) can be generated by the reaction of (**4**) with PhIO – the characteristic absorption band at 950 nm, and weak signals at 600, 450 nm were observed. Interestingly, complex (**2**) is more stable in the air atmosphere (*t*_*1/2*_ = 2.75 h) than under inert gas atmosphere (*t*_*1/2*_ is about 0.5 h) [63]. The ESI–MS spectrum of (**5**) showed peaks at *m/z* = 220 {main peak related with [(N4Py)Mn^IV^ = O]^2+^}, 240 {assigned to [(N4Py)Mn^VI^ = O(MeCN)]^2+^} and 587 {connected with [(N4Py)Mn^IV^ = O(CF_3_SO_3_)]^+^} [63]. A perpendicular mode X-band EPR spectrum of (**5**) is typical of a mononuclear, *S* = 3/2 Mn^IV^ ion [63], the spin state was confirmed using the modified NMR method [67]. Furthermore, a excited states with charge-transfer and ligand-field of (**5**) were defined using combined electronic absorption, variable-temperature magnetic circular dichroism spectroscopy and time-dependent DFT methods [68]. Chemical reactivity of (**5**) is similar to the one corresponding to manganese(IV)-oxo complex with the Bn-TPEN ligand [69].

The white solid complex [(Bn-TPEN)Mn^II^]^2+^ (**4′**) was synthesized in reaction of Mn^II^(CF_3_SO_3_)_2_·2MeCN [manganese(II) salt] with Bn-TPEN in MeCN under an inert atmosphere [26, 69]. On ESI–MS spectrum, peaks at *m*/*z* = 239 corresponding to [(Bn-TPEN)Mn^II^]^2+^ and at 627 related with [(Bn-TPEN)Mn^II^(CF_3_SO_3_)]^+^ were present [69]. Reaction (**4′**) with excess of iodosylbenzene led to the formation of a greenish-yellow complex (**5′**), with a characteristic band at 1040 nm [69]. The intermediate (*t*_1/2_ ≈ 40 min at 25 °C) was characterized by X-band EPR (the presence of signals characteristic for Mn^IV^ with spin equal 3/2 was observed); ESI–MS {[(Bn-TPEN)Mn^IV^ = O]^2+^ – signals correlate with [(Bn-TPEN)Mn^IV^ = O]^2+^ at *m*/*z* = 247 and [(Bn-TPEN)Mn^IV^ = O(CF_3_SO_3_)]^+^ at *m*/*z* = 643 were obtained}; or EXAFS techniques [Mn = O bound is about 1.69 Å, what is comparable with (**5**)] [69].

## Reactivity regulators of high-valent metal-oxo complexes

Inactive redox metal ions play a key role in controlling the reactivity of high-valent complexes with metal-oxo group in various chemical as well as enzymatic processes. Nam and Fukuzumi et al*.* [70] investigated the oxidative properties of [(N4Py)Fe^IV^ = O]^2+^ (**3**) and established that, the outer non-redox metal ion, like e.g. Sc^3+^, could significantly accelerate of the electron transfer reaction rate of the Fe^IV^ = O oxidants, even to 10^8^-fold. When the triflate scandium is present, the absorption bands due to (**3**) (*λ*_max_ = 695 nm) decrease, complexes [(N4Py)Fe^III^(O)]^+^-Sc^3+^ and [(N4Py)Fe^III^(O)]^+^-(Sc^3+^)_2_ are formed, also for the Fe^IV^/Fe^III^ pair the redox potential changes from + 0.51 to + 1.35 V (*vs* SCE) [70]. Furthermore, adding Sc^3+^ can shift the of sulfide oxygenation mechanism by (**3**) from oxygen to electron transfer [66]. Mononuclear non-heme [(N4Py)Mn^IV^ = O]^2+^ can binds scandium ions; the addition of Sc^3+^ to (**5**) changed the absorption spectrum; the band at about 940 nm characteristic for (**5**) changed to a new absorption band registered at 680 nm. During the reaction, such complexes as [(N4Py)Mn^IV^ = O]^2+^-Sc^3+^ (*t*_1/2_ ≈ 12 h at 25 °C) and [(N4Py)Mn^IV^ = O]^2+^-2Sc^3+^ (*t*_1/2_ ≈ 1 day at 25 °C) were formed, the spin state of the Mn^IV^ is equeral 3/2 [67]. In oxidation reactions, the binding of Sc^3+^ ions has a large impact on the reactivity of the Mn(IV)-oxo complex. The effect includes an approximately 2200-fold increase in the rate of the thioanisole oxidation reaction (i.e., oxygen atom transfer—OAT), but also a decrease of about 180 times in the reaction of 1,4-cyclohexadiene (e.g. hydrogen atom transfer – HAT) [67]. Thus, Fukuzumi, Pushkarm, Nam et al*.* [67] demonstrated the first example of non-heme metal-oxo complex, additionally formed with redox inactive metal ions, which has an opposite effect on the reactivity of the oxo adduct in the OAT and HAT reactions. Research on tuning the reactivity of mononuclear non-heme manganese(IV)-oxo complexes were additionally carried out by Fukuzumi, Nam et al*.* [71]. They studied the impact of triflic acid (HOTf) on Mn^IV^-oxo complexes, with both ligands (*L* = N4Py and Bn-TPEN). Reddish brown complexes of the structure [(*L*)Mn^IV^ = O]^2+^-(HOTf)_2_ were prepared in situ by addition of HOTf to [(*L*)Mn^IV^ = O]^2+^ [71]. After addition of HOTf to complex (**5**) except the original absorption band (at about 940 nm), also a new one (at 550 nm, ɛ = 540 M^−1^·cm^−1^) was generated. Related changes in UV/Vis were also observed for (**5′**) (new peak at 580 nm, ɛ = 600 M^−1^·cm^−1^ was formed) [71]. Complexes with triflic acid are relatively stable at 0 °C [*t*_1/2_ = 6 h (for a complex with N4Py ligand) and *t*_1/2_ = 1.5 h (for a complex with Bn-TPEN)]. Binding of triflic acid to the oxo group of the Mn^IV^ = O complex leads to shifts of the one-electron reduction potential towards more positive potentials and influences the inverse reactivity in OAT and HAT reactions. Comparing the complex [(*L*)Mn^IV^ = O]^2+^ with [(*L*)Mn^IV^ = O]^2+^-(HOTf)_2_, it was found that, for complexes with two additionally bound HOTf groups, the reactivity in OAT reactions increased, but in HAT reactions slowdown was detected [71].

Ce^III^ ions can reversibly bind to complexes [(N4Py)Fe^IV^ = O]^2+^, forming a compound of Fe^III^-O-Ce^IV^ type, the equilibrium state of this reaction rely on the choice of solvents, and in fact on the MeCN/water ratio [72, 73]. Based on the research, it was found that Fe^IV^ and Ce^IV^ centers have similar reduction potentials. Moreover, the equilibrium contributes to a change in the spin state of the iron, from spin equal 1 observed in [(N4Py)Fe^IV^ = O]^2+^ to spin equal 5/2 characterized for [(N4Py)Fe^III^-O-Ce^IV^(OH_2_)(NO_3_)_4_]^+^. Based on this, it can be assumed that Fe(IV)-oxo complexes may participate in multi-spin reactions [72, 73].

Iron high-valent adducts with nitride group may be present in various enzymes, for example in the nitrogenases and/or in cytochrome P450, but since they have not yet been isolated, little data is available on their oxidizing properties, or their structure. In laboratory conditions, iron complexes with the Bn-TPEN ligand were used to prepare models of iron(IV)-imido compounds [74]. Based on the conducted research it was found that the complex containing the Bn-TPEN ligand and the iron(IV)-tosylimido moiety, compared to the analogous compound, but containing the iron(IV)-oxo species in the center, shows unfortunately weaker oxidizing properties [74].

## *N*-pentadentate iron and manganese complexes – recent trends in catalysis

In the chemical industry, it is important that the properly activated catalyst not only has high selectivity, but also shows precise and controlled reactivity. Moreover, in accordance with the principles of "green chemistry" the generated waste should be as little environmentally harmful as possible [75, 76]. The use of eco-friendly oxidants like dioxygen or hydrogen peroxide in broadly understood oxidation reactions is a subject of continued attention. Iron and manganese complexes with *N*-pentadentate ligands are of great interest and are the matter of many research works, therefore only the latest results of the reactions catalyzed by Fe and Mn complexes with N4Py and Bn-TPEN ligands will be discussed in this chapter (Table 2).

**Table 2:** Selected processes catalyzed by iron and manganese complexes with N4Py or Bn-TPEN

Catalyst system/oxidizer/solvent	Reaction	Reaction products with characteristic process parameters	References
[(N4Py)Fe^II^]^2+^ (0.01 mM)/O_2_/MeCN	cyclohexanone derivatives (0.01 M) oxidation	*ɛ*-caprolactones; e.g. for cyclohexanone oxidation, with (0.15 M) benzaldehyde and/or *m*-chloroperoxybenzoic acid, yields 73% and 100%, respectively*	[77]
[(N4Py)Fe^II^]^2+^ (30 μM)/O_2_/DMSO/H_2_O	DNA cleavage	drug antitumor activity; e.g. 77 ± 4.2% of necrotic/late apoptotic SKOV-3 cells, (temp. 4, 37 °C)	[41]
[(N4Py)Fe^II^]^2+^ (0.2 mM)/plastoquinone analogs (0.50 mM)/H_2_O (0.50 M) /MeCN	photodriven water oxidation	O_2_, yield – almost 100%**	[4]
[(N4Py)Mn^II^]^2+^ (10 μM) or [(Bn-TPEN)Mn^II^]^2+^ (50 μM)/acetate buffer (50 mM)	catalytic formation of chlorine dioxide from chlorite (2–10 mM)	chlorine dioxide (for both catalysts – 31%)***	[26]
[(N4Py)Mn^II^]^2+^ (2–4 mM) /Oxone^®^ or H_2_O_2_ (10 − 250 mM)/H_2_O	oxygen evolution	O_2;_ e.g. for Oxone^®^ – V_max_ = 12.3 ± 1.7 [mol of O_2_/(mol of Mn)/h] (temp. 25 °C)	[82]
[(N4Py)Mn^IV^ = O]^2+^ (0.5 mM)/ CF_3_CH_2_OH /MeCN	cyclohexene (0.2 M), cyclooctene (0.1 M) oxidation	corresponding ketone, alcohol and/or epoxide; cyclohexenol (34%), cyclohexenone (6%), cyclohexene oxide (~ 6%) and cyclooctene oxide (91%), respectively (temp. 25 °C)	[81]
[(Bn-TPEN)Mn^II^]^2+^ (1 mM)/Al(OTf)_3_ (2 mM) /MeCN/CH_2_Cl_2_	catalytic epoxidation of cyclooctene (0.05 M)	corresponding oxide (89%), (temp. 0 °C, time: 3.5 h)	[85]
[(Bn-TPEN)Mn^II^]^2+^ (0.5–10 mM)/air or O_2_/MeCN and/or MeOH	cyclohexene (1–4 M) oxidation	corresponding ketone, alcohol, and epoxide; e.g cyclohexanone (12%), cyclohexenol (6%) and traces of epoxide^****^	[84]
[(Bn-TPEN)Mn^IV^ = O]^2+^ (0.5–0.8 mM) /TFE/MeCN	anthracene (0.10 mM), 9-methylanthracene (0.20 mM), 9,10-dimethylanthracene (0.25 mM) oxidation	corresponding ketones **–** anthraquinones; e.g. anthracene was completely (100% yield) oxidized to anthraquinone (temp. 0 °C, time: ~ 50 min.)	[86]
[(Bn-TPEN)Mn^IV^ = O]^2+^ (0.5 mM)/CF_3_CH_2_OH /MeCN	cyclohexene (0.2 M), cyclooctene (0.1 M) oxidation	corresponding ketone, alcohol and/or epoxide; cyclohexenol (26%), cyclohexanone (8%), cyclohexene oxide (18%) and cyclooctene oxide (94%), respectively (temp. 25 °C)	[81]

*The highest conversions after 15 h, at 60 °C

**Calculated on the basis of the introduced amounts of plastoquinone analogues, at 25 °C

***Calculated using the ratio of the final ClO_2_ concentration to reacted chlorite concentration; for 4 mM [ClO_2_^−^]_0_, at ambient temp

****Calculated on the basis of the introduced substrate concentration; for 1 mM catalyst in MeCN, 1 M substrate, after 24 h, under O_2_ atmosphere, at ambient temp

**Scheme 1:**
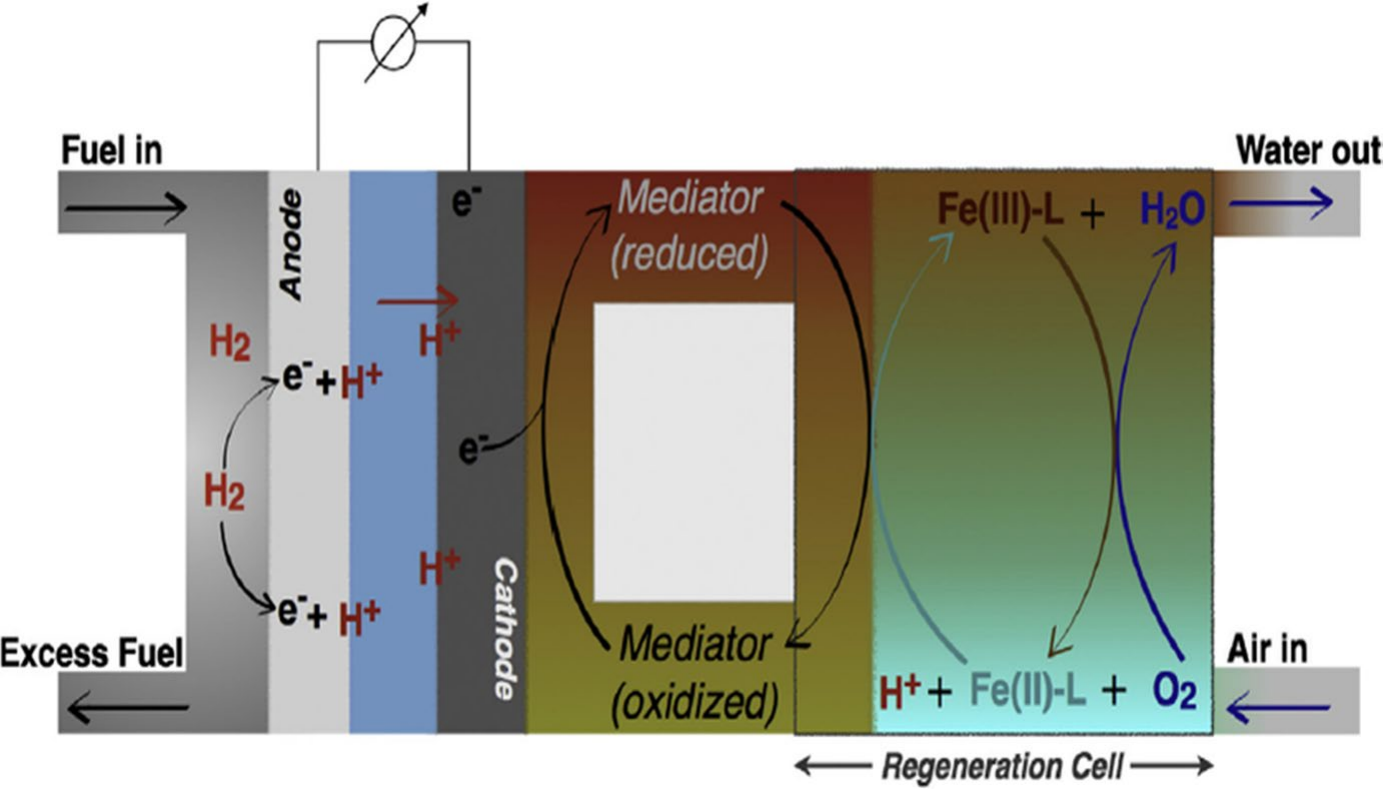
Figure presenting, in a schematic manner, the principle of work of a Proton Exchange Membrane fuel cell employing the FlowCath^®^ technology. An iron complex with *N*-pentadentate ligands like N4Py and/or Bn-TPEN, with an electron source mediator, is applied as a catalyst to reduce dioxygen, and is an alternative to the cathode dioxygen reduction reaction [78]. Reprinted from Elsevier Copyright^©^ 1969, https://doi.org/10.1016/j.jpowsour.2018.07.056, further permissions related to Scheme 1 should be directed to Elsevier

Recently iron *N*-pentadentate catalysts (**1**) were used in oxidation of cyclohexane to cyclohexanol and cyclohexanone by hydrogen peroxide [28]. The [(N4Py)Fe^II^]^2+^ complex, in the presence of aldehydes like benzaldehyde and isobutyraldehyde, as co-reductants, activates dioxygen and is able to purposefully and efficiently carry out Baeyer–Villiger reaction, in which oxidation of cyclohexanone derivatives to *ɛ*-caprolactones occurs [77], or similarly to iron(II) complexes with Bn-TPEN, can find application in chemically regenerated fuel cells as a redox catalyst (Scheme 1) [78].

In the diagram presented, a liquid phase redox mediator, that flows through the cathode and acts as an electron source is used, and iron(II)/(III) complexes with N4Py and/or Bn-TPEN are applied to the reduction of oxygen from air. Iron(II) complexes combine with the reduced form of the mediator to give iron(III) forms, which react with molecular oxygen to regenerate the iron(II) complex. Such a solution may contribute to the improvement of the functions of standard fuel cells, based on the cathodic oxygen reduction reaction. The introduction of FlowCath^®^ technology with proton exchange membrane can be a competitive method, and consist in a usage of the cathode with the liquid catalyst regeneration system, which enables a significant reduction in platinum content and is associated with lower costs [78]. Moreover, complexes [(N4Py)Fe^IV^ = O]^2+^ and [(Bn-TPEN)Fe^IV^ = O]^2+^ can replace oxygen atom in reaction with H_2_O [79], and with derivatives of *p*-benzoquinone as a plastoquinone counterpart, has been successfully used in the photodriven reaction of water oxidation [4]. Both N4Py and Bn-TPEN ligands produced highly reactive Fe^IV^ = O complexes [63], with weaker solvent-interactions, and the Mn^IV^-oxo units seems to be more basic than its Fe^IV^-oxo analogues [11]. For the Mn^IV^ = O adducts, DFT computations predicted that (**5**) has a larger barrier for H-atom abstraction from cyclohexane than (**5′**) [80]. The complexes (**5**) and (**5′**) can catalyse oxidation of olefins [81]. It is interesting that [(N4Py)Mn^II^]^2+^ shows the ability to oxidize H_2_O to O_2_ in the presence of Oxone^®^ [82, 83] or hydrogen peroxide [82], which is important for research into manganese oxidants that can be used to oxidize water. Furthermore, non-heme coordination complexes of manganese, [(N4Py)Mn^II^]^2+^ and [(Bn-TPEN)Mn^II^]^2+^ catalyze the reaction in which chlorine dioxide is obtained from chlorite, and opens the door to new method of the preparation of chlorine dioxide, which can be used on site or in the preparation of ClO_2_. They also eliminate the need for more expensive and complex higher molecular weight porphyrin ligands [26]. [(Bn-TPEN)Mn]^2+^ catalyze oxidation of cyclohexene by dioxygen to ketone and alcohol [84], and epoxidation of cyclooctene, where redox-inactive metal ions, acting as a Lewis acid in oxygen atom transfer reaction were used [85]. However, when there are no redox-inactive metal ions in the reaction medium, then the manganese(II) catalyst reacts very sluggish, (8.7% yield of epoxide), and addition of Al^3+^ ions to manganese(II) complex significantly improves epoxidation (88.9% yield of epoxide) [85]. Recent reports on the non-heme synthetic [(Bn-TPEN)Mn^IV^ = O]^2+^ complex revealed its capability to activate strong C-H bonds [86]. Manganese(IV)-oxo complexes (**5′**) and [(Bn-TPEN)Mn^IV^= O(Sc(OTf)_3_)_2_]^2+^ were used in reaction of anthracene, 9-methylanthracene and 9,10-dimethylanthracene oxidation to corresponding ketones (e.g. anthracene to anthraquinone) [86].

## Oxidative DNA cleavage

**Scheme 2:**
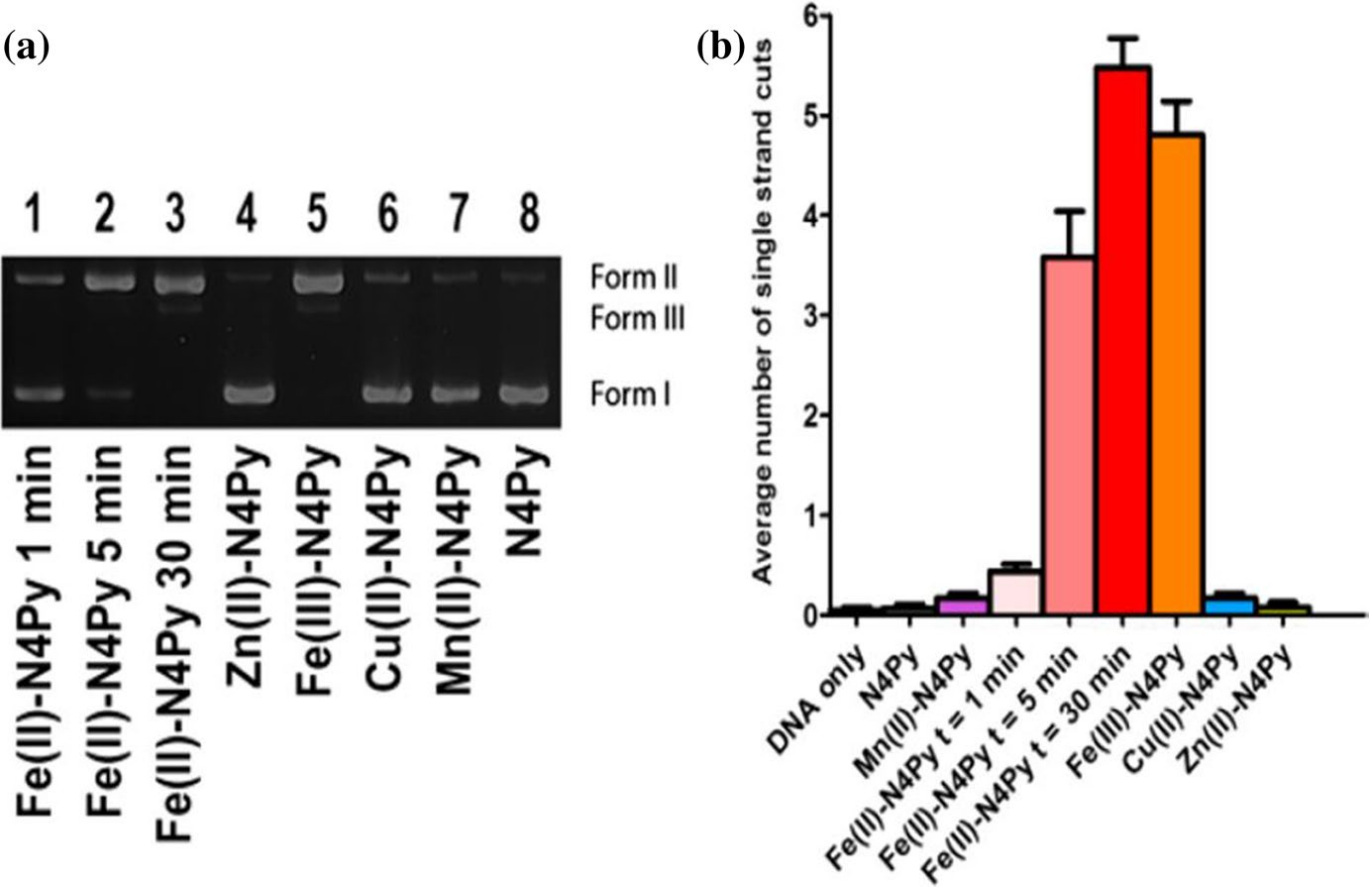
**a** Gel cleavage analysis, where 1.0 μM complex, 0.1 μg/μL pUC18 plasmid DNA, and 1.0 mM dithiothreitol (DTT) were used in TrisHCl (pH 8.0) at 37 °C. From supercoiled DNA (form I), nicked (form II) and linear (form III) DNA were obtained. After 30 min of incubation only for complexes of Fe(II) and Fe(III) with N4Py traces of supercoiled DNA were observed. **b** Mean number of single strand cleavages according to DNA particle [31]. Reprinted from American Chemical Society, Copyright^©^ 2018, https://pubs.acs.org/doi/10.1021/acs.inorgchem.8b00714, further permissions related to Scheme 2 should be directed to the ACS

In the case of iron complexes with Bn-TPEN, as well as with N4Py, studies of activity against human 20S proteasome were carried out. Both tested compounds were found to be effective inhibitors of the studied proteasome, and this information may be of use in further research on their anti-cancer activity. Moreover, it was found that iron complexes with N4Py or Bn-TPEN act according to other mechanisms, which may be respectively connecting to the enzyme or its oxidation [87]. Recently, the usage of the discussed complexes for oxidative DNA cleavage is limited to the ones based on iron, mainly supported on N4Py skeleton. Rots, Roelfes et al*.* showed that a combination of iron(II), zinc(II) or copper(II) compounds can be produced when N4Py is introduced to cell cultures, nevertheless it is possible that the metal ion will be further switched by other metal ions available in cells. In the reported research, complexes of Mn(II) ions and N4Py were also analyzed, but it was found that for coordination reasons [average length of the bonds between metal and N4Py nitrogen in the manganese(II) compounds is larger than the one in the iron(II) complexes; nevertheless the manganese(II) ions with analyzed *N*-pentadentate ligand indicate the most distorted structure] the formation of Mn(II)-N4Py is not privileged [31]. Moreover, Mn(II) ions are easily exchanged for Fe(II), yielding a stable complex [(N4Py)Fe^II^]^2+^, and only insignificant activity of manganese complexes in DNA cleavage reactions was found (Scheme 2). Also, based on a comparison of data obtained as a result of chemical and biological tests, it was suggested that (**1**) is a compound leading to oxidative destruction of cells. After the addition of free ligand (N4Py), high activity was found, which may be due to the combination of biological scavenging reactions with oxidative destruction reactions induced by the iron(II) complex [31].

The transition from the singlet state to the higher excited states is expected to occur via the triplet intermediate state, the triplet state would allow to a reaction with ^3^O_2_, which initiates DNA cleavage by oxygen activated by [(N4Py)Fe^II^]^2+^. In aqueous solution of (**1**) the possibility of transition between singlet and, probably, quintet states may be an important prerequisite for its ability to cleave DNA [33]. It was demonstrated that shortwave laser irradiation in the absence of a reducing agent can increase the activity of [(N4Py)Fe^II^]^2+^ in cutting DNA [35], which is a very important application of transition metal complexes in photodynamic therapy as agents that can be used to cleave DNA [35]. Furthermore, it was shown that in the presence of chromophores, such as 1,8-naphthalimide or 9-aminoacridine, reactive oxygen species scavengers with an additional contribution of photo irradiation can enhance the ability of (**1**) to cut DNA [42]. The research aiming at understanding the reaction mechanism showed that the crucial point in DNA oxidations reactions is the interaction of superoxide radicals with [(N4Py)Fe^II^]^2+^ to form Fe(III)-peroxo species and/or Fe(III)-hydroperoxide adducts, which are suggested to be the active complexes [43]. Such interaction, due to the ability to carry out both direct and selective DNA cleavage reactions, can contribute to the formation of more active transition metal complexes with *N*-pentadentate ligands, which may be used as synthetic compounds mimicking iron(II)-BLM.

**Scheme 3:**
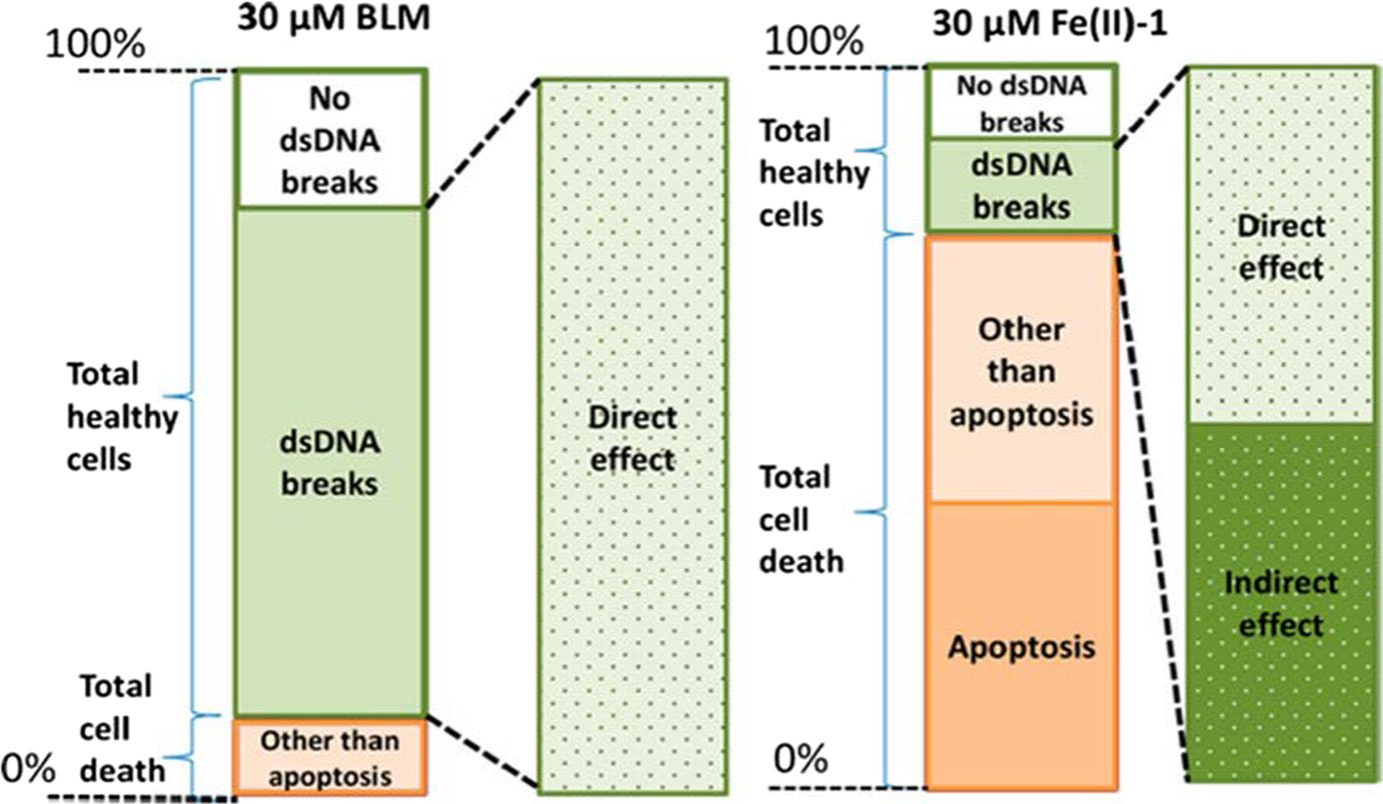
Variable effect on living cells BLM *vs* N4Py treatment. After 48 h, a different response was obtained with SKOV-3 cells treated with BLM or N4Py. 30 μM BLM causes a large number of double standard DNA (dsDNA) breaks, but more cells die upon treatment with 30 μM N4Py [41]. Reprinted from American Chemical Society, Copyright^©^ 2014, https://pubs.acs.org/doi/10.1021/cb500057n, further permissions related to Scheme 3 should be directed to the ACS

Before SARS-CoV-2, cancer was known to be one of the major causes of death, therefore new substances for chemotherapy drugs are constantly searched for. The iron carbonyl complex [(N4Py)Fe^II^(CO)]^2+^ connected with a short peptide has strong photo-induced cytotoxicity and and may find application in the treatment of prostate cancer [39]. The [(N4Py)Fe^II^(CO)]^2+^ complex is stable in an aqueous solution, has an appropriate arrangement of ligand enabling binding with peptides, also shows photo-induced toxicity towards PC-3 cancer cells. Additionally, it can strongly bind CO and is capable of rapid photolytic release of this group [39]. Moreover, application of N4Py-based iron(II) complexes in the treatment of SKOV-3 and MDA-MB-231 cancer cells showed effective double stranded DNA cleavage, similar to the effect obtained with bleomycin (Scheme 3) [41].

DNA strand breaks resulting from the application of the Fe(II)-N4Py complexes were caused by the triggering of apoptosis preceded by permanent DNA cleavage and oxidative damage to other cellular components. In addition, the ability of these complexes to directly oxidize DNA cleavage was observed [41].

## Future prospects and conclusion

Complexes transition metal ions especially iron and/or manganese have become important oxidants in industries. They catalyze the reactions of oxidation of organic compounds with dioxygen or hydrogen peroxide, and reduce the amount of environmentally harmful waste generated. Recent catalytic researches are focused on a significant increase in the efficiency of the catalyzed reactions e.g., by introduction of special reactivity regulators, which should bring benefits to industrial branches. Additionally, complexes of *N*-pentadentate ligands with iron/manganese ions show similarity to natural systems, and may find application as potential drugs. Candidates for new pharmaceuticals should have an appropriate structure for attachment to peptides – this condition is fulfilled by the discussed iron and manganese complexes with N4Py and Bn-TPEN ligands. Nevertheless iron compounds with N4Py have still proved to be more effective in cutting DNA than the corresponding manganese ones. Laser irradiation can intensify the activity of iron(II)-N4Py in cleaving DNA, which is very important for the use of transition metal complexes in photodynamic therapy, as agents that can be used to DNA cleavage. Moreover, the iron carbonyl complex [(N4Py)Fe^II^(CO)]^2+^ may find application in the treatment of prostate cancer. On the other hand, studies on the use of analogous complexes with the Bn-TPEN ligand in reactions with DNA have not been published yet, which, due to the similarity to N4Py in the way of activating dioxygen, seems to be only a matter of time. This review may contribute to the spread of new application directions for the catalysts based on the skeleton of the described *N*-pentadentate ligands, starting from the synthesis of new drugs, through photodriven water oxidation reaction, finally to their application in sustainable technologies.

## Abbreviations

N4Py: *N,N*-Bis(2-pyridylmethyl)-*N*-bis(2-pyridyl)methylamine
N4Py^*^: N,N-bis(2-pyridylmethyl)-1,2-di(2-pyridyl)ethylamine
Bn-TPEN: *N*-Benzyl-*N,N′,N′*-tris(2-pyridylmethyl)ethane-1,2-diamine
BLM: Bleomycin
PhIO: Iodosylbenzene
OTf: CF_3_SO_3_^−^
TFE: Tetrafluoroethylene
LS: Low-spin
HS: High-spin
MeCN: Acetonitrile
MeOH: Methanol
OAT: Oxygen atom transfer
HAT: Hydrogen atom transfer
